# Giant aneurysm arising from a cortical middle cerebral artery branch presenting as an extra-axial tumour: a case report

**DOI:** 10.1093/jscr/rjad210

**Published:** 2023-04-22

**Authors:** Swati Jain, Thanasis Paschalis, Tilak Das, Adel Helmy

**Affiliations:** Division of Neurosurgery, Department of Clinical Neurosciences, University of Cambridge, Cambridge, Cambridgeshire, UK; Divison of Neurosurgery, University Surgical Cluster, Singapore; Division of Neurosurgery, Department of Clinical Neurosciences, University of Cambridge, Cambridge, Cambridgeshire, UK; Division of Neurosurgery, Department of Clinical Neurosciences, University of Cambridge, Cambridge, Cambridgeshire, UK; Division of Neurosurgery, Department of Clinical Neurosciences, University of Cambridge, Cambridge, Cambridgeshire, UK

## Abstract

The size and anatomical complexity make giant intracranial aneurysms challenging surgical lesions. There is limited literature available for those arising from distal branches. The cases that have been reported in the literature have all presented with symptoms from a rupture leading to an intracranial haemorrhage. In this case report, the authors present a case of a giant aneurysm arising from a cortical branch of the middle cerebral artery presenting as an extra-axial tumour. A 76-year-old gentleman presented with a 2-day history of subjective left arm numbness. Imaging revealed a large conical right-sided parietal lesion. Intraoperatively, it was found that the lesion was being supplied by a single vascular pedicle. Histology was consistent with an aneurysm. In this case, that patient did not have any evidence of a rupture unlike all reported cases of cortical giant aneurysms. This case highlights the myriad location and presentation of giant intracranial aneurysms.

## INTRODUCTION

Intracranial giant aneurysms are defined as aneurysms with at least a diameter of 25 mm [1, 2]. With relatively dismal natural history, their size and anatomical complexity make them challenging surgical lesions. They are found in all locations with greatest propensity to be associated with the ICA (34–67%), ACA (11–40%) and MCA (13–56%). They can develop de novo or from smaller saccular aneurysms [3, 4]. They may be associated with a history of trauma, systemic arteriopathies or infections. Occasionally, these aneurysms have presented with bony erosions of the surrounding calvaria, such as the sphenoid sinus and anterior clinoid process [2, 5]. The authors present an interesting case of a thrombosed giant aneurysm arising from M4 branch with surrounding bony remodelling and erosion, raising the suspicion of this being an extra-axial tumour.

## CASE REPORT

**Figure 1:**
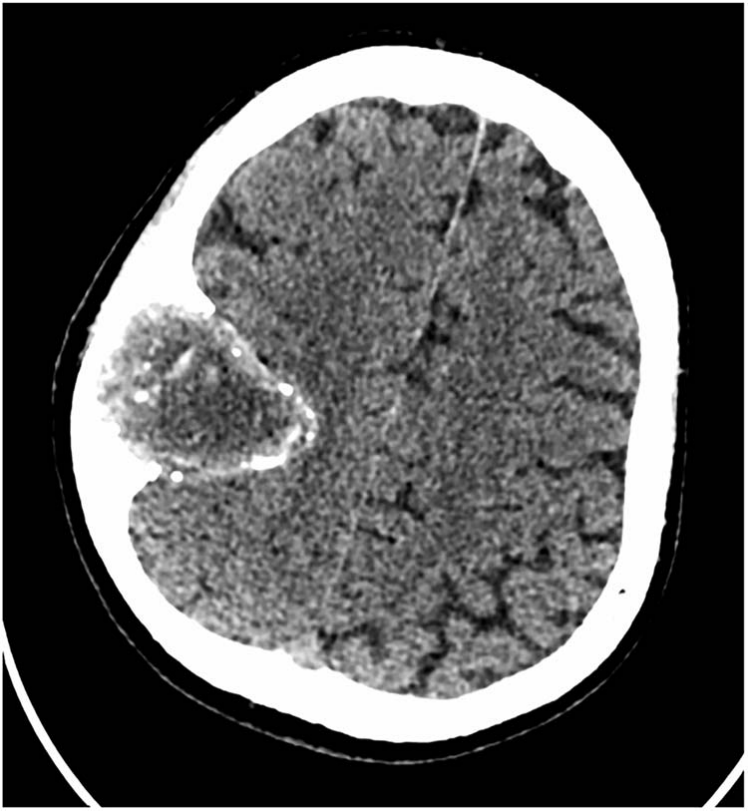
Axial CT scan showing the extra-axial lesion with surrounding bone remodelling.

**Figure 2:**
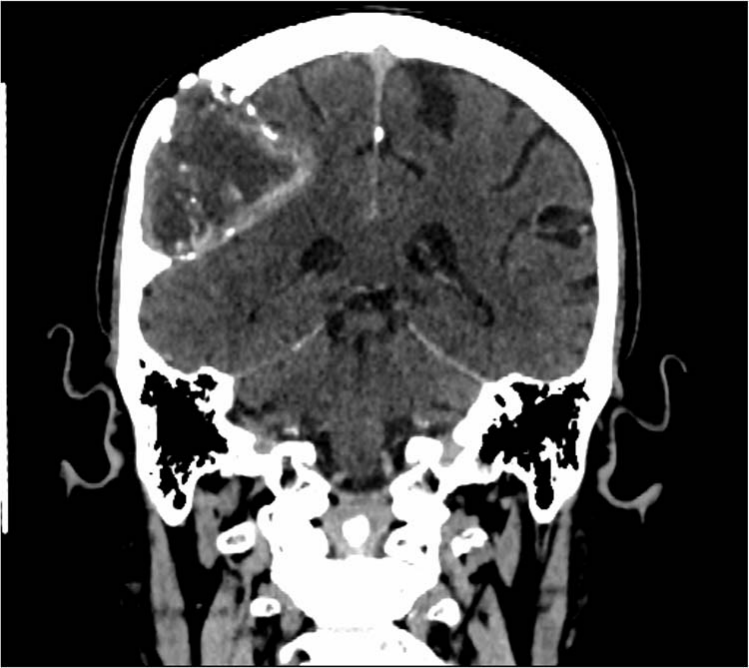
Sagittal CT scan showing the extra-axial lesion with surrounding bone remodelling and evidence of the bone erosion as well.

**Figure 3:**
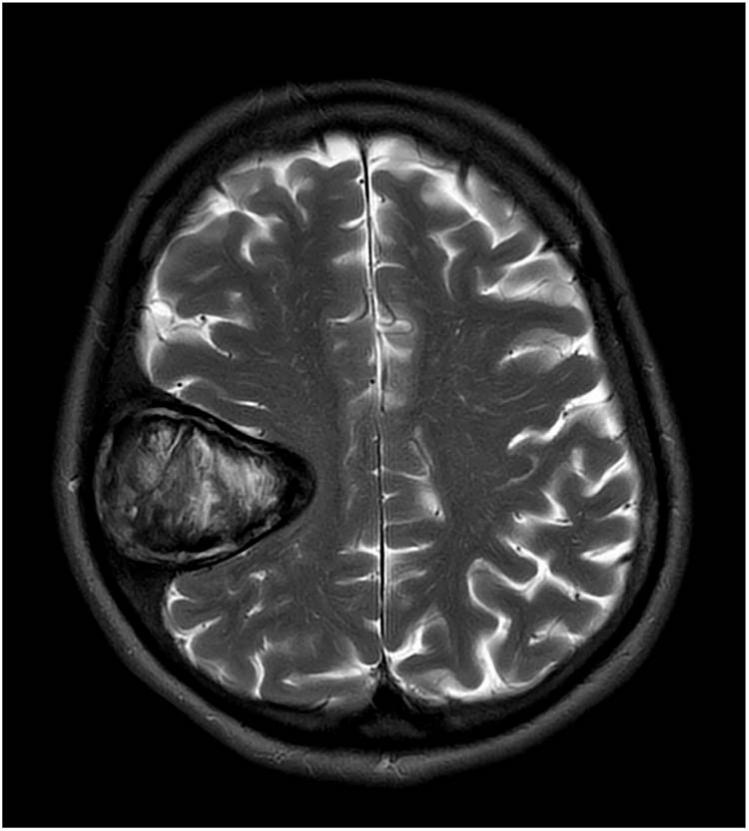
T2-weighted axial MRI scan showing the lesion with intralesional haemorrhagic products. There is no evidence of dural invasion.

**Figure 4:**
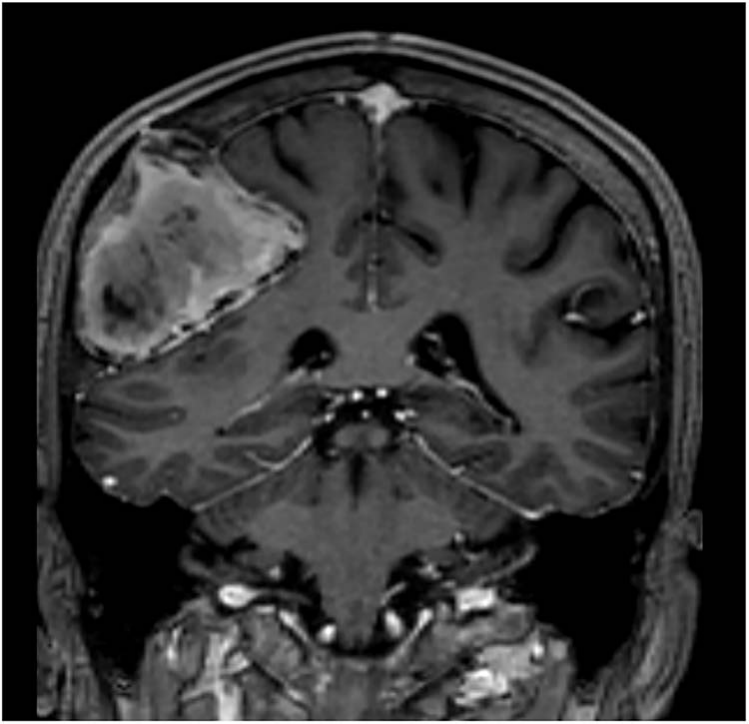
T1-weighted contrast-enhanced coronal MRI scan showing the lesion with intralesional haemorrhagic products. There is no evidence of dural invasion.

**Figure 5:**
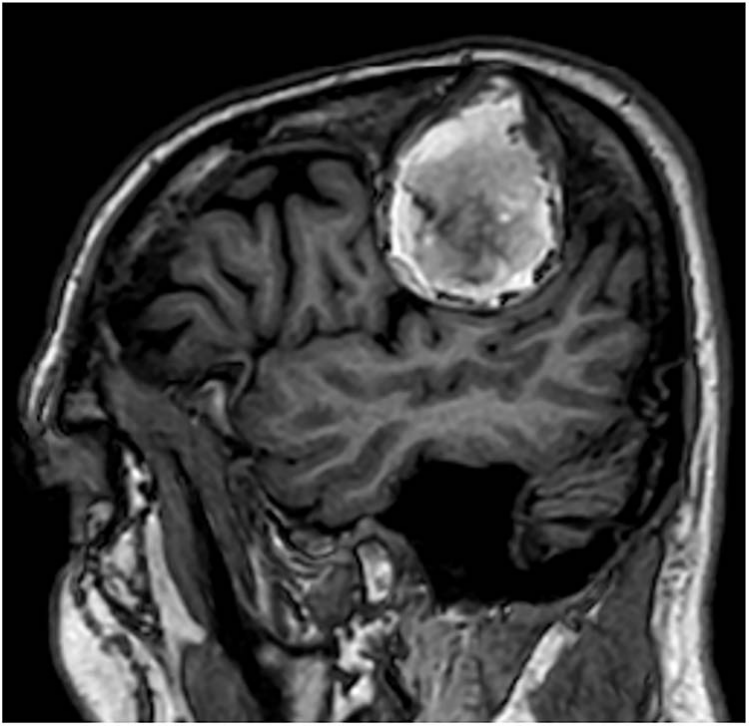
T1-weighted contrast-enhanced sagittal MRI scan showing the lesion with intralesional haemorrhagic products. There is no evidence of dural invasion.

Our patient is a 76-year-old gentleman with medical history of hypertension and lumbar spondylosis. He did not have any previous history of trauma or systemic infections. He had presented to his local hospital with a 2-day history of left arm numbness. He did not report any headaches or giddiness. On clinical assessment, he did not have any neurological deficits. A CT brain and MRI brain (Figs 1–5) with contrast revealed a 53 × 53 × 41 mm partially calcified, heterogeneously enhancing conical shaped right extra-axial parietal convexity lesion. The overlying calvarial bone was remodelled and possibly eroded. There was evidence of prior haemorrhage within the lesion and scattered internal and peripheral calcification. Based on these characteristics, the possible diagnoses were that of an intraosseous meningioma, haemangioma and giant cell tumour. Patient was consented for craniotomy and excision of the tumour.

**Figure 6:**
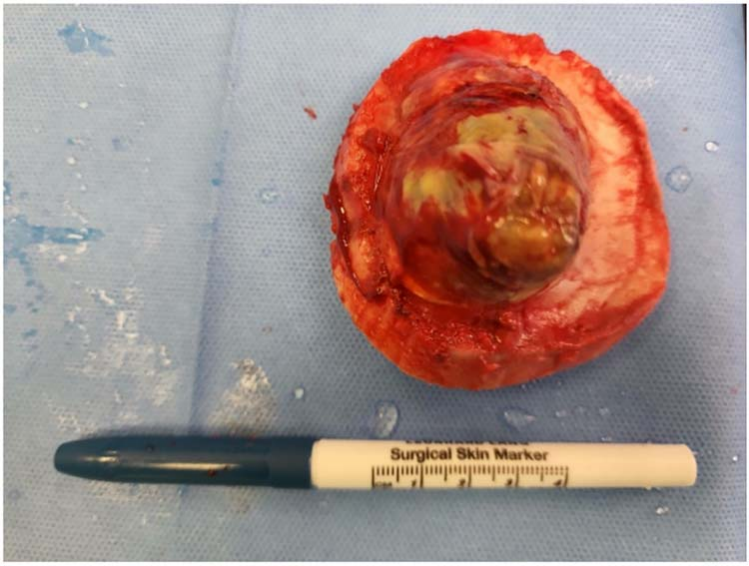
Intraoperative photo of the lesion while still attached to the bone and after the feeding vessel has been cauterized.

**Figure 7:**
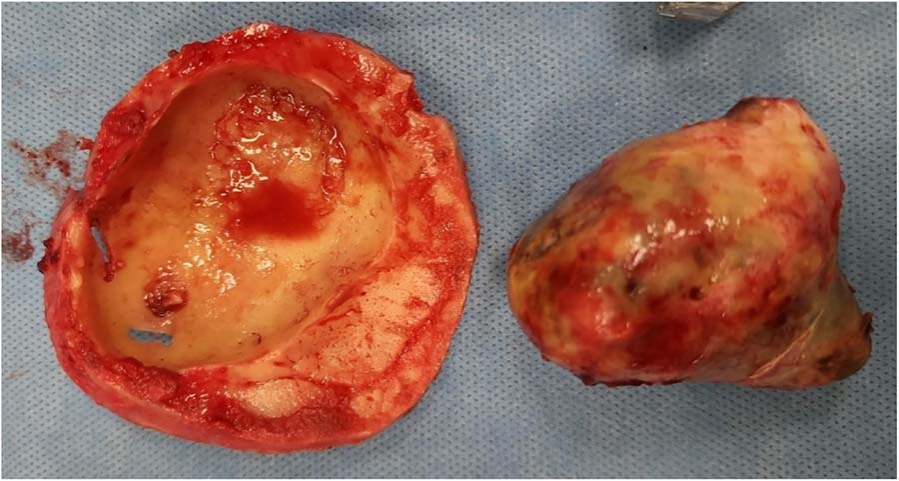
Intraoperative photo of the lesion after the lesion has been dissected off the bone. Note evidence of the bone remodelling as well as erosion of the bone.

**Figure 8:**
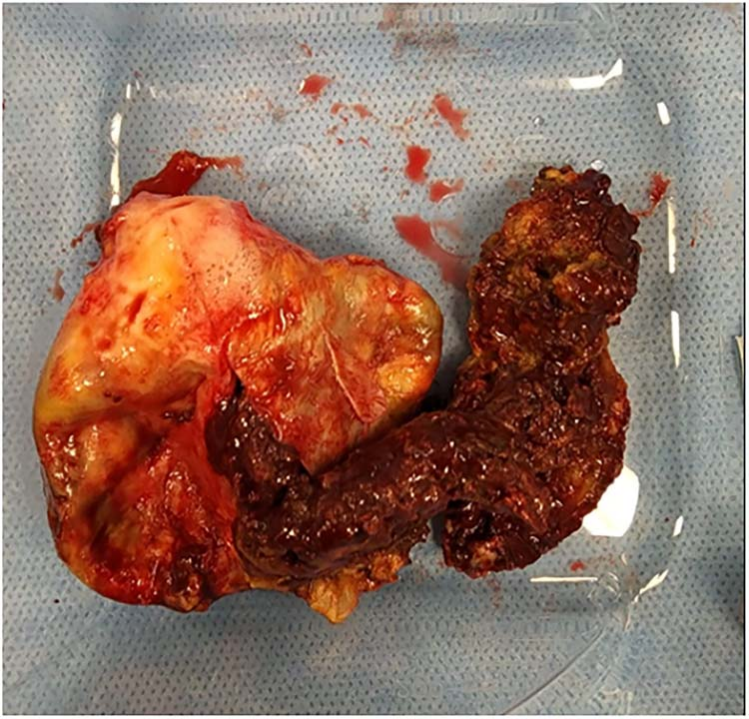
The lesion was cut open with the scalp and intralesional haemorrhagic products.

Intraoperatively, a small area of dehiscence in the bone overlying the lesion was noted. The lesion was found to be adherent to the bone but was easily dissected off the dura. There was a small dural defect at the deepest aspect of the lesion through which a vascular pedicle was noted to be supplying the lesion. The vessel was coagulated, and lesion was removed (Figs 6 and 7). The lesion was subsequently dissected off the bone and noted to have no attachment to the bone. The lesion was opened with a knife that revealed haemorrhagic products (Fig. 8). Histology revealed thickened fibrotic wall, focally lined by endothelial cells on its inner aspect. There were foci of ossification as well as areas of degenerate smooth muscle fibres (actin positive). There were haemosiderin-laden macrophages and clusters of plasma cells. These features were consistent with that of giant saccular aneurysm. Postoperatively, the patient recovered well with no neurological deficits.

## DISCUSSION

Intracranial giant aneurysms are surgical challenging entities which can be present in both anterior and posterior circulation [1, 3, 6, 7]. Interestingly, there is little data available if distal cortical aneurysms can present as giant aneurysms. Most of the MCA aneurysms occur at the bifurcation, with the cortical aneurysms being rare and often associated with history of trauma or infections such as endocarditis or bacteraemia. Reported cases of the cortical aneurysms in literature, irrespective of size, have all presented with aneurysmal rupture. Hoz *et al*. [8] recently reported a case of an idiopathic M4 giant aneurysm presenting with an intracerebral haematoma. The authors also evaluated the literature for cortical artery aneurysms which showed 14 cases that presented as subarachnoid haemorrhage or intracranial haemorrhage.

In our case, the patient was relatively asymptomatic with no evidence of ICH or SAH. We postulate this his subjective symptoms of numbness were a result of mass effect from the aneurysm on the somatosensory cortex. Recurrent thrombosis within the aneurysm would have led to progressive growth and increasing mass effect. The presence of bony remodelling and bony dehiscence further points to the chronicity of this aneurysm. The authors evaluated the reported cases of 14 giant cortical aneurysms [8–12] in the literature to identify any similar features. None of these aneurysms had evidence of bony erosion despite having similar cortical locations.

The authors expanded the literature review to find evidence of aneurysms presenting as extra-axial masses. Katayama *et al*. [7] reported a case of giant thrombosed aneurysms of the posterior cranial fossa with patient presenting with cranial nerve palsies and gait instability with progressive increase in size of the aneurysm. They could not demonstrate the arterial feeder on CT angiogram or on digital subtraction angiography. No surrounding bony erosion was reported. Fisher *et al*. [2] reported three cases of giant aneurysms presenting as extra-axial masses. These aneurysms arose from cavernous sinus with extension into middle cranial fossa and erosion of the sphenoid sinus/ethmoid sinus/parapharyngeal spaces. These showed evidence of thrombosis and rim calcification. These features are consistent with what we found in our patient.

Histologically, the aneurysm showed presence of endothelial cells and smooth muscle cells in the wall. The presence of bone remodelling around the aneurysm indicates that this aneurysm has gradually increased in size over a period. Various factors have been previously attributed to the growth of giant aneurysms [1, 3, 6, 13, 14]. These include wall shear stress resulting in endothelial dysfunction from haemodynamic stress, layering of fresh thrombus formation over chronic thrombosis from recurrent haemorrhage and neoangiogenesis from the vasa vasorum of the intracranial vessels. Given the distal location of the aneurysm, the wall shear stress is likely to have been lower as compared with the aneurysms arising from proximal locations. Given the presence of large thrombus within the aneurysm, the authors conclude that its likely recurrent haemorrhage within the aneurysm leading to its growth. There was no evidence of inflammatory cells on the histology to suggest an inflammatory cause of the growth.

Treatment of ruptured giant artery aneurysm remains tricky. Clipping, bypass and trapping, and endovascular treatment can be used for occlusion. In our case, surgical treatment was relatively easy with a single vascular pedicle that was easily coagulated.

This case highlights the myriad location and presentation of giant intracranial aneurysms. Further research is required to understand the pathophysiology of these aneurysms.
